# A Conceptual Model to be Used for Community-based Drinking-water Improvements

**Published:** 2006-09

**Authors:** Richard G. Anstiss, Mushfique Ahmed

**Affiliations:** ^1^ Faculty of Health and Environmental Sciences, Auckland University of Technology, Private Bag 92006, New Zealand; ^2^ Department of Geology and Mining, University of Rajshahi, Rajshahi 6205, Bangladesh

**Keywords:** Drinking-water, Conceptual model, Arsenic, Arsenicosis, Pathogens, Bangladesh

## Abstract

A conceptual model that can be applied to improve community-based drinking-water in crisis-type situations has been developed from the original general science and technology/development bridging concept and from a case study in Northwest Bangladesh. The main feature of this model is the strengthened role of communities in identifying and implementing appropriate drinking-water improvements with facilitation by multi-disciplinary collaborative regional agency networks. These combined representative community/regional agency networks make decisions and take actions that involve environmental and health data, related capacity factors, and appropriateness of drinking-water improvements. They also progressively link regional decisions and actions together, expanding them nationally and preferably within a sustainable national policy-umbrella. This use of the model reflects stronger community control and input with more appropriate solutions to such drinking-water crisis situations and minimization of risk from potentially-inappropriate ‘externally-imposed’ processes. The application here is not intended as a generic or complete poverty-alleviation strategy by itself but as a crisis-solving intervention, complementary to existing and developing sustainable national policies and to introduce how key principles and concepts can relate in the wider context. In terms of the Bangladesh arsenic crisis, this translates into community/regional networks in geographic regions making assessments on the appropriateness of their drinking-water configuration. Preferred improvement options are decided and acted upon in a technological framework. Options include: pond-sand filters, rainwater harvesting, dugwell, deep-protected tubewell, and shallow tubewell with treatment devices. Bedding in the regional drinking-water improvement configuration protocols then occurs. This involves establishing ongoing representative monitoring and screening, clear delineation of arsenic-contaminated wells with inter-regional linking, and national expansion within national drinking-water policy frameworks.

## INTRODUCTION

Many communities, particularly in developing countries, frequently experience serious public-health problems associated with drinking-water, including the issues of unsafe quality of water and quantity. While the community-based approach to the management of drinking-water is a popular and widely-attempted response strategy, there is a lack of translation into large-scale sustainable provision and functional integrated systems (1, 2). The mechanisms involved in developing and implementing the important issues of institutional infrastructure, technical capacity, and community participation have been summarized and highlight the need for a multi-disciplinary, collaborative, participatory and sustainable approach (1–5). Communities, however, can come to feel disconnected from broader large-scale strategic drinking-water policies and plans, particularly in times of crises. In terms of technically-related solutions, for example communities may view the need for external ‘technical promotion’ or ‘black-box’ type approaches as simply a reflection of the technology being inappropriately designed and implemented. In this context, we have developed a practical conceptual model that can be used for complementing broad water-management policies and strategies (6, 7) to improve sustainable drinking-water quality and quantity. This can be carried out through the key local-to-intermediate scale community/regional agency networks that merge on a national scale. The model is a mechanism by which there can be both improved community connection to sustainable national policies and a greater degree of community control and input. In terms of broader poverty alleviation, additional factors, such as reductions in the faecal-oral disease-transmission route that require improvements in sanitation and hygiene, will also be necessary alongside such drinking-water improvements (4).

Our long association and experiences over the years with the Maharajpur-Chapainawabganj region of Northwest Bangladesh in particular have been used for further development of the general bridging concept for the appropriate use of science and technology (2) in this model. Within these networks, the technical aspects of drinking-water improvements are integrated into community and institutional structures and are spread country-wide ideally under a sustainable national policy-framework umbrella. The model is demonstrated here in terms of Bangladesh's ongoing arsenic problem in drinking-water.

## CASE STUDY BACKGROUND

Around three decades ago in Bangladesh, a shift in drinking-water sources occurred from surface water to groundwater in tubewells. The new tubewell water was available in adequate quantities and was free from pathogens. Now, approximately 97% of the Bangladesh population use groundwater for drinking purposes through using up to as many as 10 million tubewells. There are, at present, generally five main groups of community-based water sources in Bangladesh: shallow tubewells, deep tubewells, dugwells, surface water, and rainwater.

Unfortunately, the groundwater has been found (as in West Bengal, India) to contain toxic levels of arsenic, exposing approximately 35 million people in Bangladesh alone (8). The regional arsenic exposure as it stands today has been described as the largest case of mass poisoning in history (9). The arsenic found in groundwater has a natural geological source and exists mostly in the inorganic form approximately in the ratio of 1:1: arsenate to arsenite. There are some indications that recovery should theoretically be possible for the majority of patients with arsenicosis. The groundwater in wells (shallow tubewell, deep tubewell, or dugwell) can be arsenic-contaminated. On the other hand, surface-water ponds with pond-sand filters, rainwater from protected harvesting tanks, and dugwells are generally pathogen-contaminated (sometimes also with arsenic). These latter sources are generally regarded as the more traditional and natural sources of drinking-water. During the dry season, rainwater has the additional problem of insufficient supplies from the relatively small harvesting tanks (3, 10–14).

### Maharajpur-Chapainawabganj region

The Maharajpur-Chapainawabganj region in Northwest Bangladesh contains the Chapainawabganj arsenic hotspot—a localized area with particularly high levels of arsenic contamination in its drinking-water (3). The Chapainawabganj area receives little rain in the dry season with an average annual rainfall of 1,482 mm (1986–2001) compared to the national average of 2,550 mm (14). In response to the drinking-water situation, an initial local priority was that a representative and transparent community group be established in 2003 with the capacity to decide and act in cooperation with other members of a broader informal predominantly regional (and extended international backup) network of agencies (Rajshahi University, Rajshahi Medical college, Chapainawabganj Sadar Hospital, Chapainawabganj Sadar Upazila Health Complex and Administrative Centre, Maharajpur Union Council, local manufacturers, Bangladesh; Auckland University of Technology, New Zealand). The local Maharajpur community formed a committee to oversee the situation. This committee, linked with a local NGO, Business Industry Advisory Committee (BIAC), in collaboration with the network, began the process of decision-making and taking action to improve their drinking-water situation in the region.

The next step was representative sample testing for arsenic and pathogens in drinking-water and distribution of arsenicosis. It was formally confirmed that the availability of safe drinking-water was not enough. Since there was insufficient rain during the dry season (less than 60% of the annual national average) that ruled out pond-sand filters and rainwater harvesting tanks and since it was beyond the capability of the region to explore for deep arsenic-free aquifers, the community decided to try to increase the number of simple dugwells. The committee organized villagers to renovate their unused dugwells and dig new ones. Renovations were performed in four steps: (a) removing clay bottom of well, (b) placing coarse sand at bottom of well, (c) adding lime (CaO) to well, and (d) covering top of well. Microbiological contamination of water was at levels not to be tolerated for drinking-purposes due to strong smell and taste.

Additionally, more than 50% of dugwells dried up in the dry season. As such, in the subsequent re-assessment step, dugwells were found to be an incomplete solution to the drinking-water problem because of the limited availability of water and the significant levels of microbiological contamination. The use of dugwells continued, however, for purely washing and bathing purposes when water in these was available. Therefore, it was decided that the remaining water demand (3 villages) should be filled by mitigating arsenic from tubewell water. To facilitate this, BIAC and the committee provided an income-related loan/cross-subsidisation scheme where two categories of users were defined: one category included those who could afford a cost of Tk 1,500 for an arsenic filter unit from the local manufacturer, and the other category included those who could not. The payments for the people in the first category cross-subsidised the people from the second category, meaning that the people who could not afford the arsenic filter unit would have their units provided free of charge. The re-payments for the first category were Tk 100 per month and, to date, the loans have largely been paid off. The community/network provides for ongoing technical facilitation of arsenic filter unit operations and is in the process of bedding in the overall regional drinking-water improvement configuration. This involves establishing ongoing representative monitoring of arsenic and pathogens in drinking-water, arsenicosis-screening networks with representative coverage, and clear delineation of arsenic-contaminated wells for washing purposes only. Linking with other regions and expansion are underway within the national drinking policy frameworks (6, 7, 15).

## CONCEPTUAL MODEL

An intrinsic initial requirement of the general bridging concept (2) is to have transparent community representation to act as a conduit through which decisions and information can flow in both the directions between the communities and the wider regional network. Second, the regional network needs to have the necessary technical and capacity requirements to make appropriate assessments and decisions, take actions, and/or have close and suitable linkages with external agencies that can help provide these in an ongoing fashion. For drinking-water improvements, after assessing their current configuration, each network needs to make decisions and take actions on modifications, taking into account the data on environment and health, capacity, and appropriateness. On completion of the modifications, the networks should re-assess their configuration, and if not satisfied, the process can be repeated by returning to the ‘options’ decision step. The communities satisfied with their configuration continue to the next step in the process, which involves the network bedding of the protocols. Bedding of the protocols should take place with inter-regional linkages and expansion developing from the geographically distinct local networks preferably within a sustainable national policy-framework umbrella (Fig.).

**Fig.**
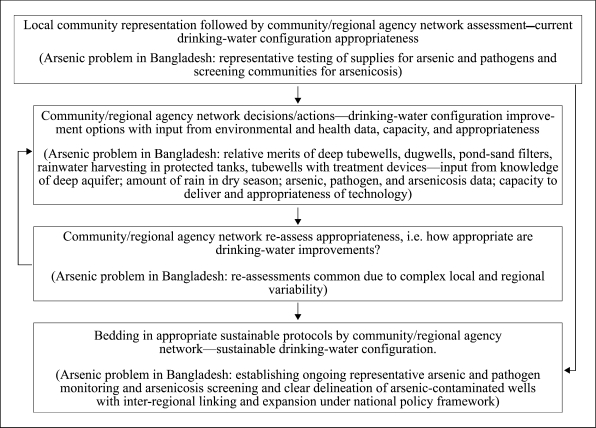
Conceptual model used for community-based drinking-water improvements. Bangladesh's arsenic example shown in parentheses

In the Bangladesh arsenic crisis situation, the overall determination of drinking-water appropriateness involves a network assessment of the existing drinking-water configuration by representative sample testing of supplies for arsenic and pathogens in relation to standard drinking-water quality parameters. It also involves representative screening of communities for arsenicosis. The networks then must decide whether their current configuration is worth bedding in as it is or whether modifications are first necessary. For the latter, a decision has to be made on what action should be taken, taking into consideration whether there is sufficient rain in the dry season (compared to the national average); whether it is known if there is a sufficient arsenic-free deep aquifer; arsenic, pathogen, and arsenicosis data; and capacity and appropriateness based on the general technical background. If there is enough rain in the dry season and enough known arsenic-free deep aquifers, each community needs to decide whether they want to improve drinking-water by increasing the numbers of traditional devices (pond-sand filters, dugwells, rainwater harvesting) and/or increasing numbers of protected deep tubewells, or first looking for arsenic-free deep aquifers if not known. The deep tubewell option tends to be relatively more favoured if there is not enough rain in the dry season; if there is no known arsenic-free deep aquifer, traditional device options tend to be favoured. If, in addition to there not being enough rain in the dry season, there is also no arsenic-free deep aquifer or one could not be found or this option was not favoured, the most technically-interventionist option left is to increase the numbers of arsenic-treatment devices on tubewells. Re-assessments of appropriateness of the drinking-water configuration should be common as there is complex local and regional variability. In Bangladesh, the process of bedding in the regional drinking-water improvement configuration protocols involves establishing ongoing representative monitoring and screening and clear delineation of arsenic-contaminated wells with inter-regional linking and national expansion within the national policy frameworks (6, 7, 15).

## DISCUSSION

While not a generic or complete poverty-alleviation strategy, the developed conceptual model introduces how key principles and concepts can relate in the wider context. It has been used here in this form for drinking-water improvements in crisis situations and incorporates important lessons from case-study experiences in Bangladesh. A point of difference from many ‘community-based’ programmes is that the model tends to be oriented towards local intermediate-scale outcomes with integration to larger-scale policy/strategy, rather than oriented towards larger-scale policy/strategy with integration to local outcomes. The orientation of this model lends itself to stronger practical community processes and control. Functional and transparent community representation is crucial in collaboration with a regional agency network that either directly or indirectly can provide the necessary appropriate technical and capacity requirements for drinking-water improvements (1–5).

A central element in the delivery of the intermediate-scale institutional infrastructure is the extent to which regional agency networks provide/facilitate appropriate and multi-disciplinary technical collaboration, connection, and capacity on the regional, national and international scales, including bridging where necessary. Intermediate-scale institutional infrastructural support could perhaps focus on helping to build these aspects of delivery. In the case of Bangladesh, aspects of this are already included in the Government's policy documents. However, the conceptual model described here adds another practical dimension to facilitate sustainable implementation of these policies (1–3, 5–7, 15).

The model strengthens community-based processes, allowing the communities to better define and implement appropriate solutions through key local-to-intermediate-scale integrated technical, community and institutional structures that merge on a national scale. This results in more community input and control and minimizes risk from potentially-inappropriate ‘externally-imposed’ processes.

## ACKNOWLEDGEMENTS

The authors particularly acknowledge input from the Maharajpur-Chapainawabganj region and the resilience of the Bangladesh people in coping with this crisis.

## References

[1] Schouten T, Moriarty P (2003). Community water, community management: from system to service in rural areas.

[2] Anstiss RG, Storey D, Overton J, Novak B (2002). Bridging science and technology with development. Contesting development: pathways to better practice; proceedings of the Third Biennial Conference of the Aotearoa New Zealand International Development Studies Network.

[3] Anstiss RG, Ahmed M, Islam S, Khan AW, Arewgoda M (2001). A sustainable community-based arsenic mitigation pilot project in Bangladesh. Int J Env Health Res.

[4] (1998). DFID guidance manual on water supply and sanitation programmes.

[5] Deverill P, Bibby S, Wedgwood A, Smout I (2002). Designing water supply and sanitation projects to meet demand in rural and peri-rural communities.

[6] Bangladesh, Government of (1998). National policy for safe water supply and sanitation.

[7] Bangladesh, Government of (2004). National policy for arsenic mitigation.

[8] Kinniburgh DG, Smedley PL, British Geological Survey (2001). Arsenic contamination of groundwater in Bangladesh.

[9] Mandal BK, Chowdhury TR, Samanta G, Basu GK, Chowdhury PP, Chanda CR (1996). Arsenic in groundwater in seven districts of West Bengal, India—the biggest arsenic calamity in the world. Curr Sci.

[10] Ahmad SA, Sayed MHSU, Hadi SA, Faruquee MH, Khan MH, Jalil MA (1999). Arsenicosis in a village in Bangladesh. Int J Environ Health Res.

[11] World Health Organization (2001). Arsenic in drinking water (revised).

[12] Jones EM (2000). Arsenic 2000: an overview of the arsenic issue in Bangladesh.

[13] Smith AH, Lingas EO, Rahman M (2000). Contamination of drinking water arsenic in Bangladesh. A public health emergency. Bull World Health Org..

[14] Jahan CS, Mazumder QH, Asaduzzaman M (2004). Long-term trend of water level in Barind area of Bangladesh: a statistical analysis for groundwater potentiality study. Bangladesh J Geology.

[15] Bux MK (2005). Bangladesh Arsenic Mitig Water Suppl Proj Newslett.

